# Designing a DNA Vaccine-based *Leishmania major* Polytope (Preliminary Report)

**Published:** 2017

**Authors:** Shabnam JEIBOUEI, Mojgan BANDEHPOUR, Bahram KAZEMI, Ali HAGHIGHI

**Affiliations:** 1. Dept. of Parasitology, School of Medicine, Shahid Beheshti University of Medical Sciences, Tehran, Iran; 2. Cellular and Molecular Biology Research Center, Shahid Beheshti University of Medical Sciences, Tehran, Iran; 3. Dept. of Biotechnology, School of Medicine, Shahid Beheshti University of Medical Sciences, Tehran, Iran

**Keywords:** DNA vaccine, Polytope, *Leishmania major*

## Abstract

**Background:**

Leishmaniasis is a neglected disease affecting millions of people worldwide. The treatment of the disease is hampered due to high cost, toxicity and the crisis of drug resistance. Polytope approaches of genetic immunization could be a strategy for prevention of infectious diseases. Furthermore, the identification of *Leishmania* genome sequence and the application of bioinformatics assist us to devise an effective vaccine’s candidate.

**Methods:**

A linear sequence from predicted epitopes of GP63, LACK and CPC antigens was designed and was optimized using online available algorithms. The synthesized sequence (LAKJB93) was ligated to pEGFP-N1 plasmid.

**Results:**

The 264bp sequence was cloned at N terminal of GFP into pEGFP_N1 expression vector and transfect into CHO cell line. Construct was efficient expressed in CHO cells.

**Conclusion:**

The protein of LAKJB93 cosnstruct was expressed in *CHO cells* successfully.

## Introduction

*Leishmania* species complete their life cycle as promastigote and amastigote forms in the sandflies vector’s midgut and the tissues of the vertebrate hosts, respectively (1). Leishmaniasis is a sort of disease having a wide range of symptoms, affects patients in various types namely cutaneous, mucocutaneous, diffuse cutaneous and visceral Leishmaniasis. The annual incidence of the disease ranges from 1.5 to 2 million people worldwide. Now some 12 million are globally infected by different types of the disease; moreover, approximately 350 million people in 98 countries are exposed to the risk of this infection (1).

Rising prevalence of leishmaniasis in most continent, reports obtained from observing patients in North America and even Australia, as well as an increasing number of coinfectious cases with HIV in Africa, The Middle East and South Europe cause the control of the disease to become a high priority for the global health. Failure to succeed in controlling the vectors and the reservoirs of *Leishmania* species, in addition to drug resistance will leave us with no choice but to find an effective vaccine candidate in order to prevent the mentioned infectious disease (2).

In recent decades, tremendous efforts have been made to develop various anti-leishmanial vaccines. Even though Leishmanization by live parasites had protective effects on vaccinated individuals in Iran and the former Soviet Union during the 1970s and the 1980s (3–5), it stopped due to chronic and severe damages to the area of vaccination, psoriasis and immunosuppression in a number of vaccinated individuals in Uzbekistan (6). Studies have shown that the inoculated live parasites cause DTH reactions in some individuals, for they have different pathogenicity (3).

The application of antigens of this parasite in providing recombinant proteins by different researchers has produced various results (6). Antigens such as KSAC, GP63, LACK, CPC, PSA2, TRYP, and PPG have been studied by researchers either separately or in combination; however, none has been highly successful so far (6–8). Furthermore, reports confirm that the usage of epitope-based vaccines yields more desired results in comparison with that of whole protein (9).

In the current study, through using protective domains of GP63, prone to minimal genetic change as time goes by (10), LACK, highly conserved amongst *Leishmania* strains (11), CPC, which develops highly protective immunity (12), and with the aid of in silico prediction, steps were taken in order to design a gene construct and its protein expression as a suitable candidate for producing adequate protective immunity.

## Materials and Methods

Three main protective *Leishmania* antigens termed Gp63, CPC and LACK were selected to design a new polytope vaccine candidate. The full sequences of each protein were obtained from GenBank *Leishmania* freidlin strains. The accession number of Gp63, CPC and LACK was XM_001681324, XM_003722109, XM_001684508 respectively. Election of CD8+ T_Cell epitopes was based on potential of the epitopes to motivate immune responses in a *Leishmania* vaccine. We initially focused on murine H2D-d-restricted epitopes of *L. major* to design this model to create a fusion protein based on three mentioned antigens having robust protective effect to activate T cell (CD8+), protective domains were selected and potent mouse T cell epitope map was determined using “T-cell prediction” server http://tools.immuneepitope.org with the highest percentile rank. In order to join the selected epitopes linkers consist of ggggs amino acids were used to conformation of the construct.

The amino acid sequence of the designed construct optimized with http://encorbio.com/server. The construct (LAKJB93) was chemically synthesized (GeneRay Company, China) and cloned between *BamH1* and *EcoR1* restriction sites of pCDNA3.1 (+) mammalian expression vector. For express confirmation, the LAKJB93 was cloned into *BglII* and *EcoRI* sites at the N-terminus of green fluorescent protein (GFP) in pEGFP-N1 reporter plasmid. GFP expression was assessed by fluorescence microscopy at 12, 24, 40 and 48 hours after transfection of the CHO cells with pEGFP-LAKJB93 and pEGFP-N1 (as positive control).

## Results

A DNA construct containing *Leishmania major* immunodominant CD8+ Tcell epitopes (H2D_d restricted) from Gp63, LACK and CPC was candidate as DNA vaccine against *Leishmania major*. The epitopes were linked with proper spacer linker amino acid.

**Fig. 1:**
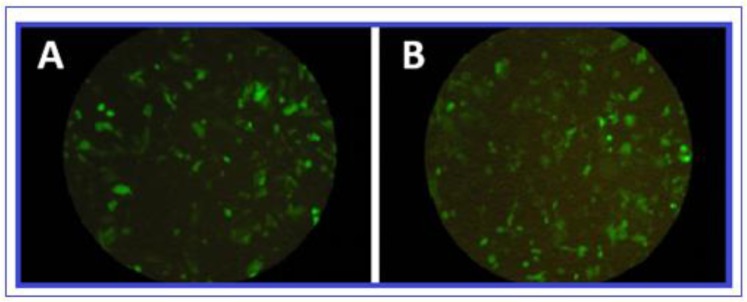
Transfected CHO cells with pEGFP_LAKJB93 (A) and pEGFP_N1 (B) after 48 hours

The 264bp sequence was chemically synthesized and cloned at N terminal of GFP into pEGFP_N1 expression vector named pEGFP-LAKJB93. PCR assay with universal primers, and sequencing analysis confirmed the cloned sequence the sequence expression in vitro was confirmed by pEGFP_N1 expression in CHO cell line. Evaluation of green fluorescent protein (GFP) expression by fluorescence microscopy at 12, 24, 48 and 72 hours after transfection showed that the construct was efficient expressed in CHO cells. The peptide expression level compared with positive control GFP expression. The best result showed after 48 hours after transfection (Fig. 1).

## Discussion

Leishmaniasis has been categorized as an uncontrolled disease by WHO. Rising resistance to conventional anti-*Leishmania* drugs along with their extensive adverse effects and low efficacy, lack of a robust vaccine together co-infection with the serious agents such as HIV and HCV, are highlighted parameters involving in inspiring Leishmaniasis (13). Hence, designing and improving a potent and efficient vaccine is one of the momentous necessities to prevent and limit of infection.

The immunity against *Leishmania* through both activation of CD4+ T cell and CD8+ T cells play a significant role in the confine the infection as well as attenuate the rate or re-infection (14, 15)13). Secretion of IL-12, IL-2, IFN-γ and TNF-α as cytokines released after activation of T helper 1 protects human against leishmaniasis. In contrast, triggering the activity of T helper 2 along with secretion of IL-4, IL-10 and IL-15 enhances the durability of infection (16–18). Therefore, it is necessary to discovery the impressive vaccine according to having potency to activate the T helper 1 and cytotoxic T lymphocyte response. Until date, despite to various studies, there is no vaccine for admitting in human (13). LACK gene, an impressive immunogen, is essential for viability of parasite thus; it has been introduced as an appropriate target for leishmaniasis therapy (19). Augmenting the plasminogen activation and plasmin formation, LACK participates in parasite invasiveness (20).

Report from a previous study pointed out the efficacy of vaccines based on *LACK* combined with other component including IL-12 and IL-18 in a murine model. Moreover, other studies revealed that DNA vaccine containing *LACK* gene could induce a potent Th_1_ immunity against leishmaniasis (21). *GP63* is another gene used in this study. This gene is conserved gene that is produced in both amastigote and promastigote lifetime. GP63 contribute to entry of parasite into itself target cell, macrophage. In addition, this protein has a proteinase properties causing increase surveillance of parasites in phagolysosomes (22). The third gene selected here is CPC is an immunogenic cysteine protease (21).

All three genes described here encode the important proteins in *Leishmania major* and support its surveillance. Selection of their robust epitope to simultaneous stimulation of CD8+ T cell immune response could be an efficient vaccine against all stages of *Leishmania major* lifetime along with having inhibitory impact on the entrance of parasite into target cells. In addition, this DNA vaccine may protect the cells by targeting proteins involved in intracellular activity of parasites. Furthermore, our findings showed that design DNA vaccine are able to express in eukaryotic cells and indicate that the designing and cloning of recombinant construct is correct and applicable.

## Conclusion

The multiepitope protein of LAKJB93 construct was expressed in CHO cells successfully.
